# Interaction of Omega, Sigma, and Theta glutathione transferases with p38b mitogenactivated protein kinase from the fruit fly, *Drosophila melanogaster*

**DOI:** 10.1093/jis/14.1.60

**Published:** 2014-01-01

**Authors:** J. Wongtrakul, K. Janphen, C. Saisawang, A.J. Ketterman

**Affiliations:** 1 Research Institute for Health Sciences (RIHES), Chiang Mai University, P.O.BOX 80 CMU, Chiang Mai, Thailand 50200; 2 Institute of Molecular Biosciences, Mahidol University, Nakhon Pathom, Thailand 73170

## Abstract

Glutathione S-transferases (GSTs) are a diverse family of phase II detoxification enzymes found in almost all organisms. Besides playing a major role in the detoxification of xenobiotic and toxic compounds, GSTs are also involved in the regulation of mitogen activated protein (MAP) kinase signal transduction by interaction with proteins in the pathway. An *in vitro* study was performed for Theta, Omega, Sigma GSTs and their interaction with MAP kinase p38b protein from the fruit fly *Drosophila melanogaster* Meigen (Diptera: Drosophilidae). The study included the effects of all five Omega class GSTs (DmGSTO1, DmGSTO2a, DmGSTO2b, DmGSTO3, DmGSTO4), all five Theta class GSTs (DmGSTT1, DmGSTT2, DmGSTT3a, DmGSTT3b, DmGSTT4), and one Sigma class glutathione transferase on the activity of *Drosophila* p38b, including the reciprocal effect of this kinase protein on glutathione transferase activity. It was found that DmGSTT2, DmGSTT3b, DmGSTO1, and DmGSTO3 activated p38b significantly. Substrate specificities of GSTs were also altered after co-incubation with p38b. Although p38b activated DmGSTO1, DmGSTO2a, and DmGSTT2, it inhibited DmGSTT3b and DmGSTO3 activity toward xenobiotic and physiological substrates tested. These results suggest a novel link between Omega and Theta GSTs with the p38b MAP kinase pathway.

## Introduction

Glutathione S-transferases (GSTs, EC 2.5.1.18) are multifunctional enzymes involved in detoxification and excretion of physiological and toxic substances ( Hayes et al. 2005 ). Several studies indicated that in addition to the enzymatic functions, GSTs also have roles such as regulation of mitogenactivated protein kinase (MAPK) pathways. A number of reports have shown that GST enzymes can interact and modulate several proteins in MAPK pathways, e.g. JNK, TRAF2, and ASK1 ( Adler et al. 1999 ; Cho et al. 2001 ; Wang et al. 2001 ; Wu et al. 2006 ; Laborde 2010 ). Mammalian GSTs have been categorized into at least seven classes; Alpha, Mu, Pi, Omega, Sigma, Theta, and Zeta ( Frova 2006 ). By extending the mammalian nomenclature, insect GSTs have been grouped into six classes: Delta, Epsilon, Omega, Sigma, Theta, and Zeta ( Enayati et al. 2005 ). Studies on the insect GSTs have mainly focused on their roles in conferring insecticide resistance, e.g., Delta and Epsilon GSTs ( Hemingway 2000 ; Enayati et al. 2005 ). The Omega, Sigma, and Theta classes have been much less studied, and their non-enzymatic roles have not been elucidated. There is accumulating evidence that suggests GSTs in Omega, Sigma, and Theta classes have important roles in oxidative stress ( Singh et al. 2001 ; Burmeister et al. 2008 ; JaramilloGutierrez et al. 2009; Piaggi et al. 2010 ; Nair and Choi 2011 ; Kim et al. 2012 ), which also suggests additional roles in cell signaling in response to oxidative stress.

Cellular behavior in response to extracellular stimuli is mediated via intracellular signaling pathways, such as the MAPK pathways. Several MAPK families have been reported, including extracellular signal-regulated kinase, Jun kinase (JNK/SAPK), and p38b MAPK ( Ono and Han 2000 ). The p38b MAPK pathway plays a major role in apoptosis, differentiation, survival, proliferation, development, inflammation, and other stress responses. The p38b MAPK has a role in a number of important diseases, such as cardio- vascular diseases and inflammatory diseases, including cancers. Therefore, modulating p38b MAPK may represent a novel therapeutic target for those diseases. This study, using a *Drosophila* model, focuses on the p38b kinase and possible interactions between GST enzymes and p38b in modulating p38b signal transduction. Several studies have found that GSTs were involved in p38b signaling. It was reported that GST mu directly interacts with ASK1 and modulates p38b MAPK signaling ( Cho et al. 2001 ; Dorion et al. 2002 ). In addition, GST mu is a regulator of dexamethasone-induced apoptosis by inhibition of Bim through down regulation of p38b MAPK and up regulation of NF-κB p50 ( Hosono et al. 2010 ). GST mu expression also has been shown to regulate p38b phosphorylation. It was found that hypertension susceptible C57BL/6 VSM cells, vascular smooth muscle cells, possessed lower levels of GSTM1 compared to VSM cells from a resistant strain and had significantly more p38b mitogenactivated protein kinase phosphorylation after H2O2 exposure ( Yang et al. 2009 ). H2O2 treatment and knockdown of GSTM1 in the resistant VSM cells also showed increased phosphorylation of p38b kinase.

Previously, we reported a GST-p38b interaction with insect specific GST Delta class, ( Wongtrakul et al. 2012 ), therefore we would like to further extend the study with Omega, Theta, and Sigma GSTs from *Drosophila melanogaster* Meigen (Diptera: Drosophilidae). The study included all five Omega class GSTs (DmGSTO1, DmGSTO2a, DmGSTO2b, DmGSTO3, DmGSTO4), all five Theta class GSTs (DmGSTT1, DmGSTT2, DmGSTT3a, DmGSTT3b, DmGSTT4), and the one Sigma class GST. Four of the GSTs are splice variants from their respective genes, DmGSTO2a and DmGSTO2b from Omega 2 gene and DmGSTT3a and DmGSTT3b from the Theta 3 gene ( Saisawang et al. 2012 ). These three classes are expressed in all known eukaryotes. Using an *in vitro* kinase assay and two different p38b substrates, ATF2 and Jun, we examined whether substrate specificity changes occurred for p38b upon GST interaction. We also studied kinase effects on GST activity toward both xenobiotic and physiological substrates. Our data showed interaction effects occurred between several GST isoforms and p38b MAPK.

## Materials and Methods

### Recombinant protein expression

The expression and purification of all proteins were carried out as previously described ( Bukhtiyarova et al. 2004 ; Udomsinprasert et al. 2004 ; Saisawang et al. 2012 ). For ATF2, a HiTrap Chelating HP (GE Healthcare, www.gehealthcare.com ) charged with NiCl2 was used according to the manufacturer’s instruction. The purified proteins were stored in 50% glycerol, 10 mM DTT, 50 mM phosphate buffer pH 6.5 for GSTs or 50 mM Tris-HCl pH 8.0 for the kinase pathway proteins at - 20°C.

### GST substrate specificity

Substrate specificity of DmGST enzymes toward five xenobiotic substrates (1-chloro-2,4- dinitrobenzene (CDNB), 1,2-dichloro-4- nitrobenzene (DCNB), ethacrynic acid (EA), p-nitrophenethyl bromide (PNPB), and pnitrobenzyl chloride (PNBC)) were measured in a 96-well plate in a spectra MR Microplate spectrophotometer (Dynex Technologies, www.dynextechnologies.com ), using the appropriate pH and λ max ( Habig et al. 1974 ). Specific activity is reported as µmol/min/mg protein.

### Effect of p38b interaction on GST activity

The effects of p38b interaction on GST activity toward some model substrates (4- hydroxynonenal (4-HNE) ( Bruns et al. 1999 ), cumene hydroperoxide (CuOOH) ( Hiratsuka et al. 1997 ), and hydroxyethyldisulfide (HED) ( Collinson and Grant 2003 )) were performed. For 4-HNE, GST activities were measured spectrophotometrically in 1-mL quartz cuvettes in a UV-visible spectrophotometer (Thermo Scientific, www.thermoscientific.com ). DmGST and p38b were desalted prior to co-incubation at room temperature using 1:1 molar ratio for 5 minutes, then GST activity was measured in comparison with the activity in the absence of p38b.

### Kinase assay

All DmGST and MAPK pathway proteins were desalted to remove DTT and glycerol prior to the kinase assay. Kinase activity assays were performed using the ADP Quest Assay (DiscoveRx, www.discoverx.com ). The p38b kinase activity was monitored using Synergy™ HT plate reader (Biotek, www.biotek.com ) operating in kinetic mode, and fluorescence intensity measurements (530 nm excitation and 590 nm emission) were made every 10 seconds for 2 minutes. Briefly, p38b (4µg) was combined using 1:1 molar ratio of GST and 100 µM ATP in ADP assay buffer. The solution was incubated for 3 minutes at room temperature before addition of 10 µg ATF2 or Jun, reagent A and reagent B, respectively. All reactions were performed in a final volume of 100 µL. Fluorescence intensity values were plotted against time to obtain a slope. The correlation between fluorescence intensity and time was linear. The background reaction rate was measured in a reaction lacking enzyme. Enzyme activities were obtained by subtracting the slope of background from the experimental sets. All experiments were performed in triplicate, and the activities were calculated using Graphpad Prism 4.0 ( www.graphpad.com ).

## Results

### Substrate profiling

**Figure 1.**
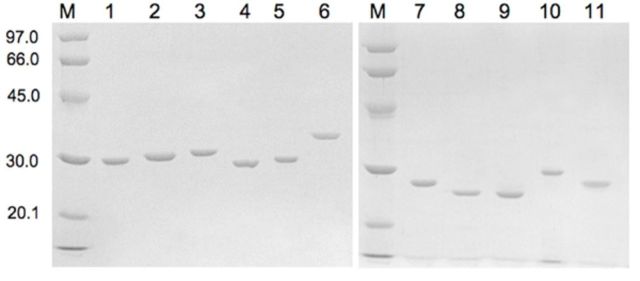
Coomassie Brilliant Blue-stained SDS-PAGE gel showing the purified recombinant proteins DmGSTTOs, DmGSTS and DmGSTTs. A Low Molecular Weight Calibration Kit was used for SDS electrophoresis (Amersham Biosciences, GE Life Science, www.gelifesciences.com ). Values are shown in kilodaltons. M: molecular weight marker; lane 1: purified DmGSTO1; lane 2: purified DmGSTO2a; lane 3: purified DmGSTO2b; lane 4: purified DmGSTO3; lane 5: DmGSTO4; lane 6: DmGSTS1; lane 7: DmGSTT1; lane 8: DmGSTT2; lane 9: DmGSTT3a; lane 10: DmGSTT3b; lane 11: DmGSTT4. High quality figures are available online.

The recombinant GSTs used in this report were purified to greater than 90% homogeneity as shown by SDS-PAGE ( Figure 1 ). GSTs display differences in substrate specificities in conjugating chemical substances. Therefore, six different substrates, CDNB, DCNB, EA, PNPB, PNBC, and CuOOH, were chosen to study for the enzymatic activities. Table 1 summarizes the results of the GST activity profiling under standard assay conditions. Only DmGSTT3b showed activity towards EA. It also had the greatest activity toward PNBC. All of the enzymes were most active with CDNB as substrate, except DmGSTT4 and DmGSTO2b, which showed no CDNB activity at the concentrations tested. Unlike other Omega DmGSTs, DmGSTO3 appears to have a broader substrate specificity displaying activity toward PNBC and PNPB. The remaining Theta enzymes also showed broader substrate specificity with the tested substrates compared to the Omega GSTs. DmGSTT3a had the greatest activity toward PNPB, which is the substrate preference of Theta class, compared to DmGSTT3b and DmGSTT1, which had 2.48-fold and 10.8- fold less activity than DmGSTT3a, respectively. Although DmGSTT1 had the lowest activity toward PNPB compared to DmGSTT3a and DmGSTT3b, it demonstrated the greatest activity for DCNB, as well as activity for CDNB. Unlike DmGSTT3a, the splice variant DmGSTT3b had detectable activity toward DCNB. DmGSTT2 can be discriminated from DmGSTT1 and DmGSTT3 by its inability to utilize PNPB. DmGSTT2 also had the ability to utilize PNBC and possessed high CDNB conjugating activity, approximately 60% of DmGSTT1. DmGSTT1, DmGSTT3a, and DmGSTT3b can also metabolize cumene hydroperoxide, suggesting possible physiological roles in oxidative stress product metabolism. The peroxidase activity of DmGSTT1 was 2-fold greater than DmGSTT3a, whereas the activity of DmGSTT3b was less than DmGSTT3a by approximately 2-fold. DmGSTT2 and DmGSTT4 had no peroxidase activity.

**Table 1.**
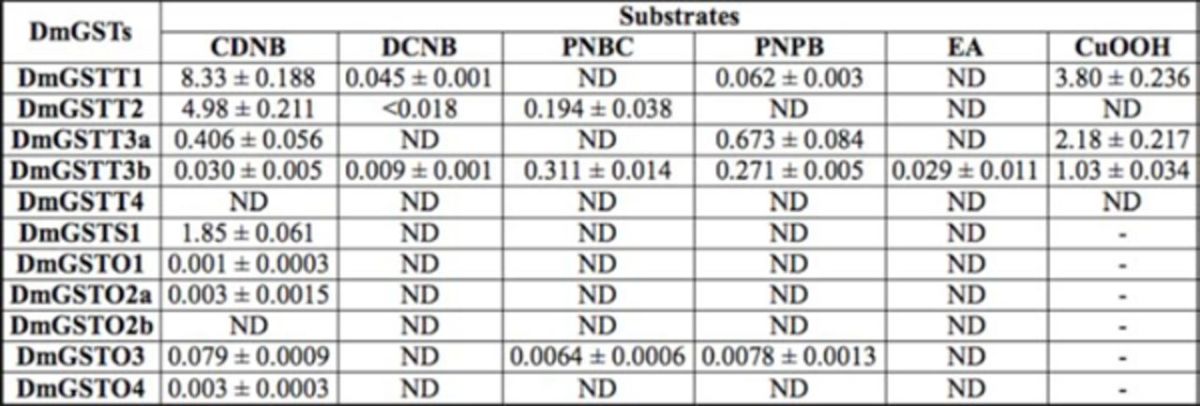
Specific activity of *Drosophila melanogaster* GSTs using various substrates.

Specific activity is shown as µmol/min/mg protein. Results are means ± SD for at least three separate assays. ND is not detected. The substrate concentrations used were: CDNB, 1 mM; DCNB, 1 mM; PNBC, 1.2 mM; PNPB, 0.1 mM; EA, 0.2 mM; and CuOOH, 1.5 mM.

### GST specific activity changes upon interaction with p38b

**Figure 2.**
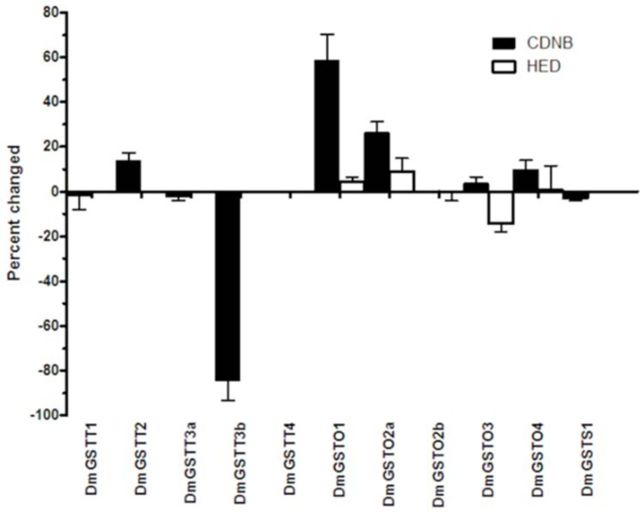
MAPK p38b changes the substrate specificities of *Drosophila melanogaster* Theta and Omega class GSTs. DmGST and p38b were incubated in 1:1 molar ratio for 5 minutes at room temperature. DmGST activity was measured using CDNB and HED as substrates. The percentage change in DmGST activity was calculated by comparing the reactions in the presence and absence of p38b. Results shown are representative of triplicate independent experiments. High quality figures are available online.

Four substrates were used to study whether p38b affected substrate specificity of GST. CDNB is a classical GST xenobiotic substrate, and three different model substrates were used: CuCOOH for general peroxidase activity, HED for thiol transferase activity, and 4- HNE, a physiological substrate originating from lipid peroxidation. For conjugating activity of GSTs toward CDNB, it was found that p38b increased DmGSTO1, DmGSTO2a, and DmGSTT2 activities 58%, 26%, and 14%, respectively ( Figure 2 ). Intriguingly, p38b decreased CDNB conjugating activity of DmGSTT3b by approximately 84%. Peroxidase and thiol transferase activities toward CuCOOH and HED in the presence and absence of p38b were furthered studied for The- Theta enzymes and Omega enzymes. It was found that peroxidase activity of Theta GSTs was changed, ranging from 0% to 7% (data not shown), whereas thiol transferase activity change ranged from 0.2% to 14% ( Figure 2 ). DmGSTO3 demonstrated the greatest percent change, approximately 14%. In addition, 4- HNE substrate, a lipid peroxidation product, was also tested with DmGSTS1. The activity of DmGSTS1 did not change upon preincubation with p38b (data not shown). Therefore, p38b showed varying effects on the GST activity for CDNB and thiol transferase activity for both Omega and Theta class GSTs.

### p38b kinase substrate specificity changes upon GST interaction

**Figure 3.**
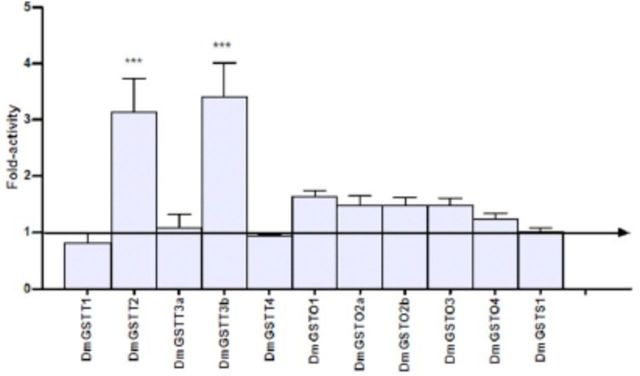
Theta, Omega, and Sigma *Drosophila melanogaster* GSTs modulate p38b kinase activity toward the substrate ATF2. The histogram shows changes in fold activity the DmGSTs had on p38b phosphorylation rates compared to the reaction without DmGSTs. The experiments were performed in triplicate. Oneway ANOVA with Dunnett’s multiple comparison test was performed with kinase reaction lacking DmGST as a control; statistical significance is shown by *** for *P <* 0.001. High quality figures are available online.

**Figure 4.**
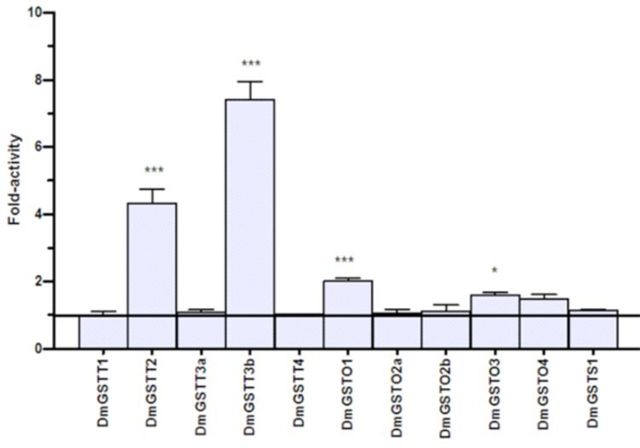
Theta, Omega, and Sigma *Drosophila melanogaster* GSTs modulate p38b kinase activity toward the substrate Jun. The histogram shows changes in fold activity the DmGSTs had on p38b phosphorylation rates compared to the reaction without DmGSTs. The experiments were performed in triplicate. Oneway ANOVA with Dunnett’s multiple comparison test was performed with kinase reaction lacking DmGST as a control; statistical significance is shown by * for *P <* 0.05 and *** for *P <* 0.001. High quality figures are available online.

Kinase activity of p38b toward ATF2 and Jun substrates in the presence and absence of *Drosophila* Theta, Omega, and Sigma GSTs was determined. For Theta class GSTs, DmGSTT1, DmGSTT3a, and DmGSTT4 did not affect kinase activity of p38b toward both ATF2 and Jun substrates ( Figures 3 and 4 ). It was found that DmGSTT2 enzyme significantly increased p38b activity toward ATF2 approximately 3.1-fold. Moreover, the enzyme also increased p38b activity toward Jun approximately 4.3-fold. Unlike DmGSTT3a, the other spliced variant of the same gene, DmGSTT3b, which possesses an extra 40 amino acids at the N-terminus, showed a 3.4- fold increase in p38b kinase activity toward ATF2. Interestingly, the interaction between DmGSTT3b and p38b also increased kinase activity for Jun 7.4-fold, which was the greatest activation of p38b observed. Kinase activity of p38b in the presence of the five Omega GSTs was slightly increased toward ATF2, ranging from 1.2 to 1.5-fold; although, none of these proteins significantly activated p38b kinase. DmGSTO2a, DmGSTO2b, and DmGSTO4 did not alter p38b activity toward Jun substrate, whereas DmGSTO1 and DmGSTO3 significantly increased p38b activity approximately 2.0 and 1.6-fold. Unlike the Theta and Omega enzymes, DmGSTS1 did not affect the p38b activity, which remained unchanged when tested with both kinase substrates.

## Discussion

Using standard GST assays, the substrate panel demonstrated that *Drosophila* Theta class GSTs had broader conjugating activities compared to the Omega class. With the exception of DmGSTT4, the enzymes tested preferred CDNB as the substrate in transferase reactions. DmGSTT1 and DmGSTT3a utilized PNPB but not PNBC, although both are reportedly Theta class GST substrates. For comparison, the mosquito AcGSTT1 utilized both PNBC and PNPB with 10-fold more activity toward PNBC compared to PNPB ( Wongtrakul et al. 2010 ). DmGSTT1 had lower PNPB activity compared to AcGSTT1 (approximately 11.8-fold less), but greater activity compared to CDNB (approximately 1.34-fold greater). In contrast, although DmGSTT1 utilized DCNB as a substrate, AcGSTT1 did not. The xenobiotic substrate selectivity of Omega and Sigma GSTs was more restricted. With the exception of DmGSTO2b and DmGSTO3, the enzymes acted only as CDNB-conjugating enzymes. Similar substrate selectivity was reported for AcGSTO1 ( Wongtrakul et al. 2010 ). The DmGSTO3 enzyme appeared to show broader substrate specificity compared to the other Omega GSTs because it showed activity towards both PNBC and PNPB, which are Theta class substrates. The GSTs in the present study appeared to show little or no peroxidase activity for the tested substrates either in this or a previous study ( Saisawang et al. 2012 ).

This appears to be in contrast to the previous reports for these GST classes and demonstrates that GST isoforms from the same class may have quite different substrate specificity ( Singh et al. 2001 ; Burmeister et al. 2008 ; Jaramillo-Gutierrez et al. 2009 ; Piaggi et al. 2010 ; Nair and Choi 2011 ; Kim et al. 2012 ).

DmGSTO1 significantly increased the activation of p38b toward Jun ( Figure 4 ). In addition, p38b also increased DmGSTO1 activity toward CDNB ( Figure 2 ). These data show that DmGSTO1 interacts with p38b, and the effects have an impact on both enzymes.

DmGSTO2a was reported to play several roles in a *Drosophila* model of Parkinson’s disease (although referred to as DmGSTO1a in the report) ( Kim et al. 2012 ). Overexpression of DmGSTO2a suppressed phenotypes caused by the *parkin* gene, resulting in loss of function, e.g., collapsed thorax, downturned wing, muscle degeneration, and dopaminergic neuron loss. The enzyme was shown to be involved in MAPK signaling, as the expression of DmGSTO2a inhibited activation of JNK in *parkin* mutants of *Drosophila* . The overexpressed DmGSTO2a blocked the activation of JNK and apoptosis in the *parkin* mutants, which also protected against the degeneration of indirect flight muscle. In our results, both DmGSTO2a and DmGSTO2b appeared not to interact with p38b for either ATF2 or Jun, suggesting that these GSTs were not involved with the p38b pathway at this level.

We observed that DmGSTO3 significantly increased p38b activity toward Jun ( Figure 4 ). In addition, p38b also decreased DmGSTO3 thiol transferase activity by approximately 15% ( Figure 2 ). This observation suggests the specific interactions of both proteins, the kinase and the GST, may affect the respective physiological roles. In adult flies, the DmGSTO3 transcript was strongly expressed in the regions associated with digestion and detoxification, namely the crop, gut, and tubules ( Walters et al. 2009 ). In embryo, expression of DmGSTO3 was only detected in stages 13–16, the stages of gut development. Although reportedly involved with oxidative stress, the DmGSTO3 shows little or no activity for oxidative stress substrates. A Fox-like transcription factor binding motif is found in proximity to the DmGSTO3 gene, and this binding motif is associated with signaling pathway and cell fate decisions in development ( Li et al. 2008 ). Down regulation of DmGSTO3 in *dcr-2* mutant *Drosophila* flies appeared to correlate with decreased survival under oxidative stress as well as a decreased life span ( Lim et al. 2011 ). This raises the possibility that DmGSTO3 may have similar roles as the human GSTO1. Human GSTO1 has been shown to modulate ryanodine receptors, which are intracellular calcium channels ( Dulhunty et al. 2001 ). It was shown some of the modulation was dependent upon Omega enzyme thiol activity. Omega enzyme activity was also found to be important for glutathionylation of ATP synthase β subunit for activity of F1F0-ATP synthase activity ( Kim et al. 2012 ). These data show the Omega GSTs possess a substrate/protein interaction specificity extending beyond p38b kinase interactions, as well as each Omega GST showing unique preferences.

**Figure 5.**
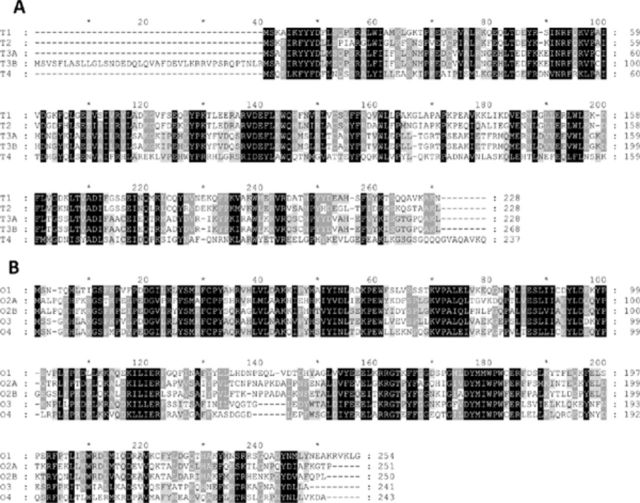
Amino acid sequence alignment of Theta and Omega GSTs. The upper panel **A** shows the four Theta GSTs aligned, and the lower panel **B** shows the five Omega GSTs aligned. Gaps introduced to maximize sequence similarity are shown by a horizontal dash. Shading indicates degree of conservation of residue, where black is 100% conserved, dark grey is > 80% conserved, and light grey is 60–80% conserved. The figure was produced by GeneDoc version 2.5. High quality figures are available online.

Comparing DmGSTT1, DmGSTT2, DmGSTT3a, and DmGSTT3b showed an amino acid identity ranging from 40% to 85% and an amino acid similarity ranging from 58% to 85% ( Figure 5 ). Accordingly, these isoenzymes have distinct substrate profiles ( Table 1 ). DmGSTT1 had substrate preferences for CDNB and cumene hydroperoxide. DmGSTT2 had no detectable activity with PNPB, which is a highly specific substrate for Theta GST, whereas both DmGSTT3a and DmGSTT3b had high activity. This is most likely due to the differences in their hydrophobic substrate binding site (H-site) conformations. It was found that p38b affected DmGSTT3b activity by reducing the CDNB activity 80% while showing no effect on GST activity for HED ( Figure 2 ). This suggests the binding of p38b induces conformational changes in the GST that differentially affect substrate specificity. Similar effects for kinase-GST interactions were also reported for JNK decreasing activity for several studied GSTs toward CDNB, DCNB, and PNBC ( Udomsinprasert et al. 2004 ). In contrast, DmGSTT3b activated p38b kinase activity significantly toward both ATF2 and Jun substrates compared to the control (Figures 3 and 4). A possible region of GST-p38b interaction might be the N-terminus of GST, as DmGSTT3b had 40 amino acids extra at the N-terminus compared to DmGSTT3a, with the remainder of the two proteins being identical ( Figure 5 ). The extra amino acids of DmGSTT3b increased p38b activity compared to the DmGSTT3a-p38b interaction 3.1- and 6.9-fold for ATF2 and Jun, respectively. This suggests that the N-terminus of GST may be involved in p38b interaction, which also has been reported for a Delta class GST. DmGSTD11a has a 21 amino acid extension at the N-terminus, which significantly affected p38b activity ( Wongtrakul et al. 2012 ). Evidence for an additional N-terminus role is that DmGSTT3b activity can be detected for DCNB, PNBC, and EA, whereas no detectable activity was observed for DmGSTT3a ( Table 1 ). Our study also suggests a role for GSTT2 in MAPK cell signaling ( Figures 3 and 4 ). DmGSTT2 similarly affected p38b activity, increasing it toward both ATF2 and Jun substrates. The mechanisms for the activation of p38b by DmGSTT2, as well as the DmGSTT2 CDNB conjugating activity increases induced by p38b, remain to be elucidated. Alignments of the four sequences of the GSTs that interacted with p38b, T2, T3b, O1, and O3, and those from the same GST class that appeared to not affect p38b activity, did not yield any clues as to the amino acids that were involved in the proteinprotein interactions ( Figure 5 ). The interactions therefore must be determined by tertiary changes that occur with the sequence variations between the enzymes.

In summary, employing several substrates for p38b MAPK as well as for DmGSTs, we observed that GSTs from Theta and Omega, but not Sigma, classes demonstrated protein interactions with p38b. These interactions increased p38b kinase activity toward its transcription factor substrates ATF2 and Jun. This has been reported previously for several Delta class GSTs interacting with p38b and showing reciprocal effects on substrate specificity ( Wongtrakul et al. 2012 ). The p38b-GST protein interactions would cause conformational changes affecting substrate preference for both the GST and the p38b kinase. The physiological functions of these interactions are areas for future research.

## Abbreviations

CDNB: 1-chloro-2,4-dinitrobenzene
DCNB: 1,2-dichloro-4-nitrobenzene
EA: ethacrynic acid
GST: glutathione S-transferases
HED: hydroxyethyldisulfide
JNK: Jun kinase
MAPK: mitogen activated protein kinase
PNBC: p-nitrobenzyl chloride
PNPB: p-nitrophenethyl bromide
4-HNE: 4-hydroxynonenal
